# Supplementary material for: A randomized trial of at-home COVID-19 tests, telemedicine, and rapid prescription delivery for immunocompromised individuals
Source: Res Sq. 2024 Oct 28:rs.3.rs-5314583. Preprint. [Version 1] doi: 10.21203/rs.3.rs-5314583/v1 (PMC11581110; doi:10.21203/rs.3.rs-5314583/v1)
Supplement: Supplement 1 [file NIHPPRS5314583V1-supplement-1.pdf]

Supplementary Files

This is a list of supplementary files associated with this preprint. Click to download.

- suppCueOct22.docx
